# Large-Eddy BreakUp Devices – a 40 Years Perspective from a Stockholm Horizon

**DOI:** 10.1007/s10494-018-9908-4

**Published:** 2018-04-11

**Authors:** P. Henrik Alfredsson, Ramis Örlü

**Affiliations:** Linné FLOW Centre, KTH Mechanics, SE-100 44 Stockholm, Sweden

**Keywords:** Turbulence, Drag reduction, Drag measurement, Skin friction, Momentum-loss calculation

## Abstract

In the beginning of the 1980’s Large Eddy BreakUp (LEBU) devices, thin plates or airfoils mounted in the outer part of turbulent boundary layers, were shown to be able to change the turbulent structure and intermittency as well as reduce turbulent skin friction. In some wind-tunnel studies it was also claimed that a net drag reduction was obtained, i.e. the reduction in skin-friction drag was larger than the drag on the devices. However, towing-tank experiments with a flat plate at high Reynolds numbers as well as with an axisymmetric body showed no net reduction, but instead an increase in total drag. Recent large-eddy simulations have explored the effect of LEBUs on the turbulent boundary layer and evaluations of the total drag show similar results as in the towing tank experiments. Despite these negative results in terms of net drag reduction, LEBUs manipulate the boundary layer in an interesting way which explains why they still attract some interest. The reason for the positive results in the wind-tunnel studies as compared to drag measurements are discussed here, although no definite answer for the differences can be given.

## Preamble

Today (in 2018) there is a strong move towards energy efficiency both when it comes to conversion of various energy sources (fossil, bio, wind, hydro, solar, etc.) into electric energy as well as efficient energy utilisation in various technical applications (heating, lighting, transportation in air, on the ground or water, etc). Also in the early 1970’s the first oil crisis made a large impact on research related to efficient energy usage especially with respect to transportation. One of the major issues dealt with was the possibility to reduce drag on vehicles, and then especially drag produced by turbulent flow over bodies. This was at least partly due to two different findings, *i)* the observation of so called coherent structures in turbulent flows [1], especially in boundary layers [2] and *ii)* the finding that very small amounts of certain polymers added to water could reduce the pressure drop in pipe flows [3]. The coherent structures leading to or being the results of so called bursts in the near-wall region of a boundary layer were found to account for more than 80% of the contribution to the turbulence production term during only 20% of the time. This gave the idea that if those structures could be manipulated it would be possible to achieve turbulent drag reduction as e.g. envisioned in the review by Liepmann (1979) [4]: “*Probably the most important aspect of the existence of deterministic structures in turbulent flow is the possibility of turbulence control by direct interference with these large structures. Such control could lead to very significant technological advances*”. Most of the earlier studies [2, 5–8], without explicitly attempting at control, focussed therefore on the detection and description of turbulence-generating events. More than twenty years earlier Toms [9] observed drag reduction in water pipe flows by the addition of small amounts of polymers. These results hinted that there were possibilities to find efficient control methods with rather small effort and in Ref. [3] the so called Virk’s asymptote for maximum polymer drag reduction was proposed which indicated up to 80% pressure drop decrease in turbulent pipe flow with only very small amounts of added polymers (10 − 50 ppm).

These interesting results gave rise to several workshops and symposia devoted to drag reduction. IUTAM symposia were arranged in Bangalore and Zürich in 1987 and 1989 [10, 11], respectively, with the titles “Turbulence Management and Relaminarisation” and “Structure of Turbulence and Drag Reduction”. Another series of workshops that is still active is the European Drag Reduction Meeting for which the first meeting was held at the Swiss Federal Institute of Technology in Lausanne (EPFL) in September 1986 [12]. This meeting was followed by several others under the auspices of ERCOFTAC and its Special Interest Group SIG20, Drag Reduction and Flow Control, which also was one of the founding groups of ERCOFTAC. The present paper is based on a talk that was recently given by the authors at the latest meeting in that series, namely the 12th European Drag Reduction and Flow Control Meeting held in Rome in April 2017. At the meeting in Rome our talk was the only one about LEBUs, whereas at the meeting in 1986 about 10 papers were devoted to outer-layer devices (LEBUs) and “more than fifty” groups where active in those years [12], so the interest in these has clearly diminished over the years. Today the general consensus is probably that LEBUs do not work for drag reduction, although other applications have been suggested.

## Introduction

In the present paper we will give a perspective based on research done over the last 40 years, research that has dealt with so called Large-Eddy Break-Up (LEBU) devices. The perspective is slightly negatively biased from our Stockholm perspective, since KTH researchers never found evidence of drag reduction in either towing tank experiments in the 1980’s [13, 14], nor 30 years later in recent Large Eddy Simulations (LES) of modelled LEBUs [15, 16].

The LEBU strand of fluid dynamic research may be said to have started in 1977 with the work by Yajnik and Acharya [17], who introduced a screen across a turbulent boundary layer (TBL) with the aim to destroy large coherent structures observed in experiments. They also observed a decrease in the skin friction downstream the screen. However, the reduction of skin friction downstream an object in the TBL is not a surprise but the question was raised if it was possible to have a net drag reduction, i.e. if the total skin friction reduction could become larger than the added drag on the inserted object.

**Fig. 1:**
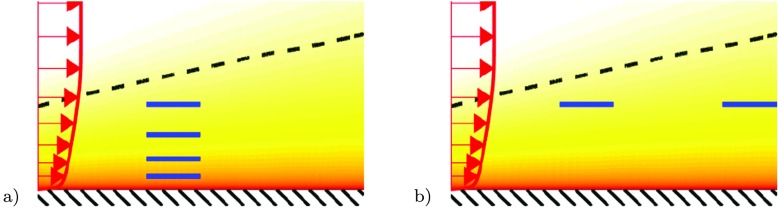
Schematic of LEBU configurations. **a** Stack of thin plates, **b** a tandem pair of LEBUs. Dashed line and colour contours indicate the boundary layer thickness and the mean streamwise velocity, respectively

The hope that such a device would work for drag reduction on bodies seemed to become a reality with the work of Corke et al. at Illinois Institute of Tecchnology (IIT) in Chicago [18, 19]. In the first study they used a stack of thin plates stretching spanwise in the boundary layer (Fig. 1a) and they observed effects both on the large scale structures and a reduction of skin friction, however, no net drag reduction was obtained. In Ref. [19] they instead used two thin blades mounted in tandem which were placed at a distance from the wall of about 80% of the boundary layer thickness (Fig. 1b) and reported a total drag reduction of 20% through measurements of the momentum-loss thickness far downstream. This result spurred a lot of interest all over the world and maybe up to 50 research groups started to work in the area [12, 20], several workshop and conference sessions were dedicated to research around LEBUs and in many of them drag reduction was reported.

**Fig. 2:**
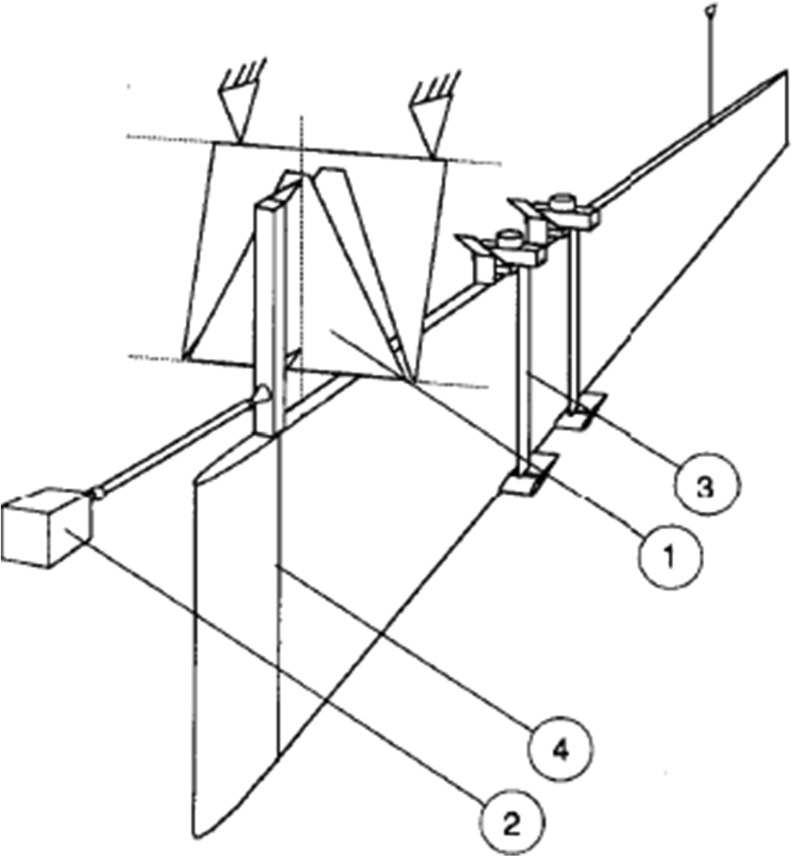
The 8.2 m long plate used by Sahlin et al. [14] for towing tank experiments. The plate width (depth) was 1 m. Note that the drag on each of the LEBU pairs could be measured individually, since they were freely suspended with respect to the plate. The normal distance from the plate could also also be varied during the run, in that way many combinations could be tested.  is the support of the plate, is the force gauge, shows the upstream LEBU with the traversing device at the top of the plate and the position of the trip wire. Reprinted from Ref. [14], with the permission of AIP Publishing

However, it was not until the towing-tank experiments carried out by KTH researchers [13, 14] which showed no net drag reduction on a long flat plate equipped with a tandem set of manipulators that the interest started to fade. The first set of experiments used and reported in Ref. [13] was first regarded with scepticism since it was on odds with the overall belief that net drag reduction had been shown earlier. Therefore, the KTH group undertook a second campaign where the plate was made longer to reach an even higher Reynolds number, 36 million based on plate length, (8.2 m, maximum speed 5 m/s as compared with 6.2 m and maximum speed of 4 m/s in the first campaign) and where the position normal to the wall of the LEBUs could be varied and also the drag on the LEBUs themselves could be measured independently (Fig. 2). However, still no net drag reduction was obtained for any of the parameter combinations tried despite the fact that the measured drag on the LEBUs was what could be expected for a well-behaved airfoil, i.e. one without vibrations or separation. Similar experiments in a towing tank at NASA with an axisymmetric body were later made and reported by Anders [21] in 1989 and these experiments also showed no drag reduction. The very detailed and accurate measurements by Lynn et al. [22] made in Germany in a wind tunnel reported in 1995, where both the plate drag and the LEBU drag were measured, also investigated many different parameter combinations, including single and tandem manipulators. Also those experiments showed the same result, i.e. no net drag reduction, where the single manipulator was found to give the lowest drag increase. After these studies interest in LEBUs for drag reduction declined, although the initial hype left its imprint in some textbooks [23], while more recent ones acknowledge that there is no documented net drag reduction when accounting for the device drag [24].

For work during the 1980’s the reviews of Bandyopadhyay [25] and Anders [26] are useful resources, among other references [27, 28]. The first of these [25] shows the status around 1985 of many different types of turbulent drag reduction attempts including LEBU devices, or what is sometimes called OLD (Outer Layer Devices). At the time of these reviews the conclusion with respect to LEBU devices was that a 10% drag reduction could be obtained. The later review by Anders [26] appeared in 1989 and at that time it was stated “*..., but reliable net drag reducing devices still remain somewhat elusive*”. That review contains 65 references where about 55 deal directly with LEBU research. Of these only a few are published in refereed journals, the others are from conference proceedings, internal reports, MSc or PhD thesis reports. Of the journal publications one is about negative results [14] whereas the others that are published in journals are of more general character, i.e. discussing structural changes and skin-friction reductions without claiming drag reduction [29]. Hence the notion that LEBUs could produce drag reduction never entered the reviewed scientific literature; maybe a good sign that journal editors actually act as gatekeepers of scientific rigour.

One reason for us to write this short review is the recently renewed interest in LEBUs since it has been possible to set up LEBU flow cases using Direct Numerical Simulations (DNS) [30, 31] to investigate the effect in more detail. While the Reynolds numbers affordable via DNS are low, the Reynolds numbers and (in particular streamwise) domain size are sufficiently large when utilising well-resolved Large Eddy Simulations [15, 16] to match some of the low Reynolds number wind-tunnel experiments. All of these studies simulated a single manipulator and they show skin-friction reduction downstream of the device although only Ref. [15] analyses the effect on the total drag. Also in that case no net drag reduction is obtained thereby confirming the direct drag measurements in towing tanks [14, 32] and in a wind tunnel [22].

One of the unanswered questions is why the momentum-loss method, which is an indirect method to evaluate the drag, seems to fail for the evaluation of drag for LEBU manipulated boundary layers. In this paper we will discuss some possible answers to this question which may also have bearings on other flow situations. We will also mention some other possible applications of LEBUs where the structural changes of the boundary layer are used. Finally we discuss the possibility of turbulent drag reduction in general, however, already at this point we would like to clarify that we believe that the future for any dramatic improvements seem to be bleak.

## How to Determine Drag?

The drag on a body moving through a fluid can be divided into form drag (pressure drag), friction drag and wave drag. The latter is the drag experienced by ships that are creating surface waves on a water surface. For aircraft travelling at transonic or supersonic speeds another mechanism creates drag, also referred to as wave drag. This drag mechanism arises from pressure differences only occurring at speeds around or above the speed of sound, however this is of no concern to us here.

There are in principle two different methods to determine the total drag on a body: the momentum method and the drag method. The drag method is a direct method, i.e. one measures the drag on a body with a force balance. Such measurements can be done in a wind or water (or other liquid) tunnel on a stationary object or in a towing tank where the body is moved at a specified speed and the drag balance is connected between the body and the pulling platform. The body can be of different forms, both a fully immersed body or a body that is partially submerged and is penetrating the surface.

**Fig. 3:**
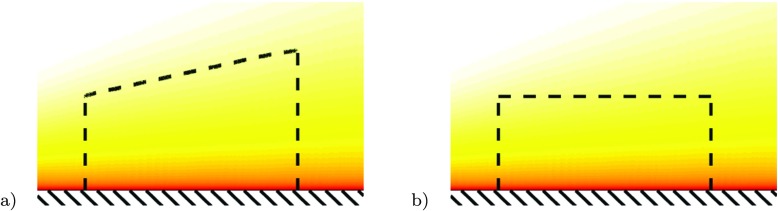
Schematic of control volumes to determine change in momentum flux. **a** Control volume where the upper surface is a streamline, **b** rectangular control volume with flow through the upper side of the CV

The momentum method is in principle a method using the momentum balance over a control volume. The choice of control volume is arbitrary, however two choices are common as depicted in Fig. 3. One is using a streamtube volume, i.e. a volume that is bounded by inflow and outflow areas normal to the incoming flow, connected by the surface off the body and a stream surface, i.e. a surface consisting of streamlines, hence no flow is passing through that surface. The second possibility is to use a rectangular control volume. In the following we will discuss these two choices.

### Stream-tube control volume

#### The von Kármán momentum integral equation

For a two dimensional case the stream-tube control volume reduces to a simple surface as seen in Fig. 3a. This model is the one used in the von Kármán momentum integral equation for laminar and incompressible flow and it can be written

1 $$ \frac{\tau_{w}}{\rho_{e}{U_{e}^{2}}}=\frac{\mathrm{d}\delta_{2}}{{\textrm d}x} +\frac{1}{U_{e}}\frac{\mathrm{d}U_{e}}{{\textrm d}x}(\delta_{1}+ 2\delta_{2})\,, $$

where *τ*_*w*_ is the local wall shear stress, *ρ*_*e*_ and *U*_*e*_ are the density and velocity outside the boundary layer, respectively. *δ*_1_ and *δ*_2_ are the displacement and momentum loss thicknesses, respectively which are defined as

2 $$ \delta_{1}={\int}_{0}^{\delta}\left( 1-\frac{U}{U_{e}}\right)\mathrm{d}y \;\;\;{\textrm{and }} \;\;\; \delta_{2}={\int}_{0}^{\delta}\frac{U}{U_{e}}\left( 1-\frac{U}{U_{e}}\right)\mathrm{d}y\,, $$

where *U* is the mean streamwise velocity. In the case of constant external flow speed (and hence a constant external pressure) it reduces to

3 $$ \frac{\tau_{w}}{\rho_{e}{U_{e}^{2}}}=\frac{\mathrm{d}\delta_{2}}{{\textrm d}x}\,, $$

and the skin-friction drag per unit width between positions *x*_1_ and *x*_2_ can be determined as

4 $$ D_{\mathrm{f}}={\int}_{x_{1}}^{x_{2}}\tau_{w}\mathrm{d}x=\rho_{e}{U_{e}^{2}}[\delta_{2}(x_{2})-\delta_{2}(x_{1})]\,. $$

If a two-dimensional body with drag *D*_body_ per unit width is introduced into the control volume the total drag per unit width (*D*_total_) will instead become

5 $$ D_{\text{total}}=D_{\mathrm{f}}+D_{\text{body}}=\rho_{e}{U_{e}^{2}}[\delta_{2}(x_{2})-\delta_{2}(x_{1})]\,,  $$

under the assumption that the flow still is laminar and there are no pressure variations along any of the control volume surfaces, which is the common boundary layer approximation.

For a turbulent boundary layer one has to take some further caution when using the Kármán integral equation since also the turbulent stresses at the inflow and outflow boundaries come into play and the equation becomes:

6 $$ \frac{\overline{\tau_{w}}}{\rho_{e}{U_{e}^{2}}}=\frac{\mathrm{d}\delta_{2}}{{\textrm d}x}- \frac{1}{{U_{e}^{2}}}\frac{\mathrm{d}}{{\textrm d}x}{\int}_{0}^{\delta} \left( \overline{u^{'2}} -\overline{v^{'2}}\right)\mathrm{d}y\,,  $$

where *u*^′^ and *v*^′^ are the fluctuating components of the streamwise and wall-normal velocity components, and the overbar indicates a time average. Note that this equation is given in [33] but with the wrong sign for the turbulence term. That error has spread to several other papers later on, and the error was later noted by the original authors [34]. The first part of the integral term on the right-hand side comes from the streamwise momentum of the turbulent fluctuations, whereas the second part arises due to the pressure variations in the normal direction. For a ZPG turbulent boundary layer the integral itself is positive since $\overline {u^{'2}} >\overline {v^{'2}}$ everywhere, and also the *x*-derivate is positive (mainly because *δ* increases with *x*). If the term is neglected one would obtain a too high shear stress. Also this equation can be integrated to obtain the skin-friction drag as:

7 $$ D_{\mathrm{f}}(x_{2}-x_{1})={\int}_{x_{1}}^{x_{2}}\overline{\tau_{w}}\mathrm{d}x=\rho_{e}{U_{e}^{2}}[\delta_{2}(x_{2})-\delta_{2}(x_{1})] - \rho_{e}\left[{\int}_{0}^{\delta} \left( \overline{u^{'2}} -\overline{v^{'2}}\right)\mathrm{d}y\right]_{x_{1}}^{x_{2}}\,. $$

In most experimental studies using the momentum equation the inflow boundary (*x*_1_) is upstream the object in the uniform free stream which then results in the following simplified equation

8 $$ D_{\mathrm{f}}(x_{2})=\rho_{e}{U_{e}^{2}}\delta_{2}(x_{2})\left[ 1 - \frac{1}{\delta_{2}(x_{2})}{\int}_{0}^{\delta(x_{2})} \frac{\overline{u^{'2}} -\overline{v^{'2}}}{{U_{e}^{2}}}\mathrm{d}y\right]\,.  $$

In experiments the integral is usually not measured and thereby not taken into account, but would give a negative contribution to the overall drag since $\overline {u^{'2}}>\overline {v^{'2}}$ everywhere inside the boundary layer. From available experimental and numerical data (obtained at low Reynolds numbers) the integral can be estimated to be of the order of 0.02*δ*_2_ [35], hence not taking it into account would overestimate the skin-friction drag by 2%.

#### The general momentum equation

**Fig. 4:**
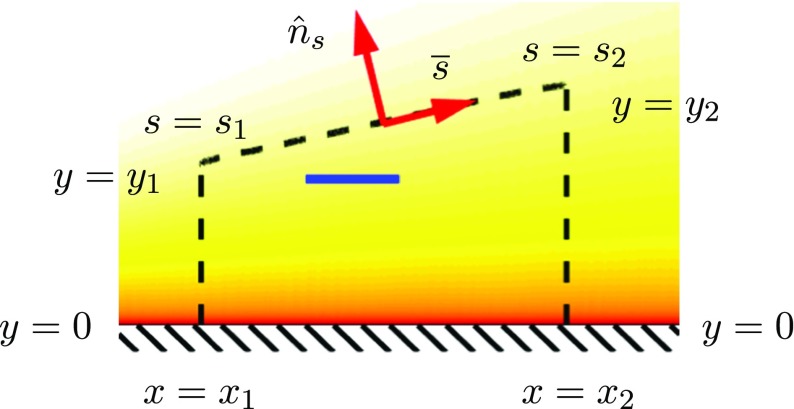
Definition of control volume and coordinate system used for Eq. 9

The results shown in Eqs. 6 and 8 above are valid for a constant free-stream velocity and hence a constant pressure on the upper stream surface that defines the control volume. This may be a good approximation for a carefully executed wind-tunnel experiment over a flat plate where the pressure gradient has been carefully adjusted to be zero. However, when inserting an object, such as a LEBU into the boundary layer this may not be true any longer. In a general two-dimensional case with a 2D-body with drag *D*_body_ per unit span immersed in the control volume the corresponding momentum balance can be expressed as

9 $$\begin{array}{@{}rcl@{}} D_{\text{body}} +{\int}_{x_{1}}^{x_{2}}\overline{\tau_{w}}dx&=&\\ {\int}_{0}^{y_{1}}\left( \overline{p_{1}}+\rho \overline{{u_{1}^{2}}}\right)dy &-& {\int}_{0}^{y_{2}}\left( \overline{p_{2}}+\rho \overline{{u_{2}^{2}}}\right)dy -{\int}_{s_{1}}^{s_{2}} \overline{p(s)}\hat{n}_{s} \cdot d\overline{s}\,, \end{array} $$

where $u_{i}=U_{i}+u^{\prime }_{i}$, *p*_*i*_ is the mean pressure and where index *i* = 1,2 refers to the upper and downstream vertical sides of the control volume, respectively. *p*(*s*) is the pressure and *s* the coordinate along the enclosing streamline, whereas $\hat {n}_{s}$ is the outward normal to that streamline, see Fig. 4. In the case that the mean pressure is constant everywhere we recover Eq. 5.

It is also well known that the skin-friction in a nominally two-dimensional boundary layer may not be homogeneous in the spanwise direction and that introducing a manipulator may severely affect the spanwise distribution (see figures 6 and 8 in Ref. [22]).

### Rectangular control volume

For a rectangular control volume the situation is in principle more simple since there is no pressure force that needs to be taken into account on the upper surface, since any resulting pressure force on that surface is normal to the drag force. However instead one needs to take into account the amount of momentum that flows out of that surface, giving the following expression for the drag

10 $$ D_{\text{total}}={{\int}_{0}^{h}}(\overline{p_{1}}+\rho \overline{{u_{1}^{2}}})dy-{{\int}_{0}^{h}}(\overline{p_{2}}+\rho \overline{{u_{2}^{2}}})dy - {\int}_{x_{1}}^{x_{2}}\rho \overline{v(x,h)u(x,h)}dx\,. $$

Note that *u* = *U* + *u*^′^ and *v* = *V* + *v*^′^, i.e. the non-linear terms will give rise to Reynolds stress terms. The last term includes the normal velocity at the upper boundary of the control volume and this probably excludes the possibility to use this control volume approach in an experiment since the accuracy of the wall normal velocity will probably be too low to obtain sufficiently accurate results. For simulations on the other hand this should be possible and was the control volume chosen in Ref. [15], which yielded zero net drag reduction in accordance with the drag-method experiments.

## Discussion

In August 1984 the EUROMECH 181 Colloquium “Drag reduction through boundary layer control” was organised in Saltsjöbaden near Stockholm where one of the themes was “Drag reduction in turbulent boundary layers – Outer layer modifications”. The first author attended together with A.V. Johansson and presented astudy “Preliminary towing tank experiments with large eddy break up devices”. Those experiments were done with aracing kayak equipped with LEBU devices but only drag increase could be reported. This study was aprecursor to the later experiments by Sahlin et al. [13, 14]. In the conclusion paragraph of the Colloquium report [36] it was stated:

*“In the discussions most speakers showed optimism concerning the possibility of reducing drag through boundary layer control, using either riblets, turbulence manipulators or surface perforations. This in spite of the fact that barely any indisputable reduction could be presented at the meeting. In order to obtain net drag reduction it seems necessary to proceed the understanding of underlying phenomena much better, and this is particularly important when extrapolating results from laboratory experiments to full scale conditions.”*

However, all results published in refereed journals indicate that the hope that LEBUs should be able to reduce turbulent friction drag at high Reynolds numbers to a larger extent than the added device drag is futile. An interesting question is why the direct drag experiments as well as the recent LES give different results compared to the momentum balance calculations obtained in wind-tunnel experiments. Note that all positive results on drag reduction are based on indirect drag measurements including the momentum balance calculations (see e.g. Table 1 in Ref. [26]). The momentum balance is used in many situations to determine for instance drag on two-dimensional bodies [37] such as wing sections by measuring the velocity distribution in the wake as well as for standard turbulent boundary layers on flat plates. The present paper presents some aspects of such an analysis but the effects discussed seem to be too small to explain the large discrepancies observed between drag results and momentum balance results for the LEBU case. This discrepancy is still unexplained and from a basic fluid dynamic point of view it could clearly be good if a satisfactory resolution would be obtained.

In a short report [38] from the 5th European Drag Reduction Meeting in London in 1990 it was stated that “*the skin friction drag reduction of LEBU devices are depressing compared to that of riblets*”. In the same meeting a possible explanation of the difference between the early wind-tunnel experiments and the results from the towing tank experiments was suggested by K.-S. Choi [38] “*... work on LEBU devices which show that a measure of three-dimensionality develops from such devices...... He suggested that if this is the case then measurements at the centreline of such devices would give a false impression.*” A spanwise non-homogeneous distribution of the effects of LEBUs may very well exist as was also shown in Ref. [22]. It should also be noted that even in standard turbulent boundary layers on flat plates in wind tunnels three-dimensionalities develop (with both smooth and rough surfaces) even if care is taken to make the flow two-dimensional [39]. In order to find the total drag in a flat plate boundary layer one would hence need to make spanwise traverses of the boundary layer in order to find out the total effect of the LEBUs. In a towing tank experiment on the other hand the total drag is measured and the result is independent on how the drag is distributed.

LEBU devices may on the other hand be useful in other contexts. One possibility is that LEBUs could reduce optical distortions when viewing through a turbulent boundary layer by decreasing the intermittency in the outer portion of the boundary layer [40]. Another possibility is to use LEBUs to reduce the pressure fluctuations at the wall by destroying at least part of the large-scale structures and thereby decreasing the induced noise, particularly annoying for airplane travel [31].

The failure of LEBUs despite its effects on the turbulence structures has not hindered other attempts to reduce turbulent drag, but then by modifying the wall boundary condition. Riblets are a rather simple approach and have been shown to give drag reduction up to 10% under ideal conditions (i.e. optimal spacing, height and geometry, main flow aligned with the riblets). This method has probably more to do with a change of the boundary condition than change of the wall turbulence *per se*. Also compliant coatings have been suggested to decrease turbulent wall friction. Maybe the most reliable results has been presented in Ref. [41] where drag reduction up to 7% was obtained in a water tunnel by direct drag measurements on a long axisymmetrical body. In addition to the drag reduction the results also showed a decrease in the fluctuations of both skin friction and wall pressure. Other attempts to reduce turbulent skin-friction drag have been to make the wall surface activate spanwise travelling waves (e.g. [42]) or spanwise wall oscillations (e.g. [43]), both methods of which have shown to be able to give skin friction reduction for certain parameters ranges. However, these two latter systems need external energy input to drive the system and when taking that into account net drag reduction is questionable. Also from a practical point of view it is at present hard to phantom how such systems should be implemented in practical applications. On the other hand drag-reduction control through delayed transition with passive methods such as miniature vortex generators (MVG, e.g. Ref. [44]) or separation control by plasma actuators (e.g. Ref. [45]) have shown potential as possible methods for future applications.

There exists a vast literature of active flow control published during the last 10-15 years, mainly in the form of numerical simulations of unstable and/or transitional laminar flows but also on fully turbulent wall-bounded flows. Such control needs both sensors and actuators, but also control schemes. Although the hype of LEBU research was based on experimental shortcomings, the active flow control area has mainly attracted applied mathematicians. However, they have shown little interest in practical flow control, and it has mainly been an exercise in theoretical/numerical manipulation of the equation of motions trying out various control schemes and the possibility to reduce turbulent drag by active control methods in practical applications seems still to be farfetched. One of few such experiment that has been reported is the one in Ref. [46] where the streaky structures in a laminar boundary layer excited by free-stream turbulence could be controlled by local blowing and suction in a reactive manner where wall mounted hot-wires were used as sensors.

It is also worth noting that the huge amount of research published about coherent structures in wall turbulence since the 1960’s, whether these structures are called *streaks, hairpins, horseshoes, arcs, attached eddies, bulges, packets of hairpins, worms, whorls, whirls, shear layers, bursts, ejections, sweeps, large-scale motions, very-large scale motions, superstructures* or something else, have not resulted in the hoped insight to manipulate turbulent boundary layers to yield net drag reduction. In that sense the optimism expressed at the EUROMECH Colloquium 181 more than 30 years ago, namely that “*the understanding of underlying phenomena*” would be helpful to reduce drag through boundary layer control has not been realised. Access to the three-dimensional and time-resolved data from recent simulations might in that sense be valuable to further illuminate the here unanswered questions, to avoid similar unnecessary hypes and guide future control strategies.

## References

[1] Brown GL, Roshko A (1974). On density effects and large structure in turbulent mixing layers. J. Fluid Mech..

[2] Kline SJ, Reynolds WC, Schraub FA, Runstadler PW (1967). The structure of turbulent boundary layers. J. Fluid Mech..

[3] Virk PS, Mickley HS, Smith KA (1970). The ultimate asymptote and mean flow structure in Toms phenomenon. J. Appl. Mech. - ASME.

[4] Liepmann HW (1979). The rise and fall of ideas in turbulence. Am. Sci..

[5] Alfredsson PH, Johansson AV (1984). On the detection of turbulence-generating events. J. Fluid Mech..

[6] Blackwelder RF, Kaplan RE (1976). On the wall structure of the turbulent boundary layer. J. Fluid Mech..

[7] Kim HT, Kline SJ, Reynolds WC (1971). The production of turbulence near a smooth wall in a turbulent boundary layer. J. Fluid Mech..

[8] Rao KN, Narasimha R, Narayanan MAB (1971). The ’bursting’ phenomenon in a turbulent boundary layer. J. Fluid Mech..

[9] Toms, B.A.: Some observations on the flow of linear polymer solutions through straight tubes at large Reynolds numbers. In: Proc. First Int. Congr. Rheology, vol. II, pp 135–141 (1948)

[10] Gyr, A. (ed.): Structure of turbulence and drag reduction. Springer), Zürich (1989)

[11] Liepmann, H.W., Narasimha, R. (eds.): Turbulence management and Relaminarisation. Springer), Bangalore (1987)

[12] Savill AM, Truong TV, Ryhming IL (1988). Turbulent drag reduction by passive means: a review and report of the first European drag reduction meeting. J. Theor. Appl. Mech..

[13] Sahlin A, Alfredsson PH, Johansson AV (1986). Direct drag measurements for a flat plate with passive boundary layer manipulators. Phys. Fluids.

[14] Sahlin A, Johansson AV, Alfredsson PH (1988). On the possibility of drag reduction by outer layer manipulators in turbulent boundary layers. Phys. Fluids.

[15] Chin C, Örlü R, Monty J, Hutchins N, Ooi A, Schlatter P (2017). Simulation of a large-eddy-break-up device (LEBU) in a moderate Reynolds number turbulent boundary layer. Flow Turbul. Combust..

[16] Chin C, Örlü R, Schlatter P, Monty J, Hutchins N (2017). Influence of a large-eddy-breakup-device on the turbulent interface of boundary layers. Flow Turbul. Combust..

[17] Yajnik, K.S., Acharya, M., Fiedler, H: Non-Equilibrium effects in a turbulent boundary layer due to the destruction of large eddies. In: Structure and mechanisms of turbulence i. lecture notes in physics, vol. 75. Springer, Berlin (1978)

[18] Corke, T.C., Guezennec, Y.G., Nagib, H.M.: Modification in drag of turbulent boundary layers resulting from manipulation of large-scale structures. NASA CR 3444 (1981)

[19] Corke, T.C., Nagib, H.M., Guezennec, Y.G.: A new view on origin role and manipulation of large scales in turbulent boundary layers NASA CR 165861 (1982)

[20] Wilkinson SP, Anders JB, Lazos BS, Bushnell DM (1988). Turbulent drag reduction research at NASA Langley: progress and plans. Int. J. Heat and Fluid Flow.

[21] Anders, J.B.: LEBU Drag reduction in high Reynolds number boundary layers AIAA-89-1011 (1989)

[22] Lynn TB, Bechert DW, Gerich DA (1995). Direct drag measurements in a turbulent flat-plate boundary layer with turbulence manipulators. Exp. Fluids.

[23] Gad-el-Hak M (2006). Flow control: passive, active, and reactive flow management.

[24] Tardu S (2017). Wall turbulence control.

[25] Bandyopadhyay PR (1986). Mean flow in turbulent boundary layers disturbed to alter skin friction. J. Fluids Eng..

[26] Anders, J.B.: Outer-layer manipulators for turbulent drag reduction. In: Bushnell, D.M., Hefner, J.N. (eds.) Viscous drag reduction in boundary layers, progress in astronautics and aeronautics, pp. 263-284 (1989)

[27] Bushnell, D.M.: Recent turbulent drag reduction research at Langley research center. NASA TM 78688 (1978)

[28] Savill, A.M.: Drag Reduction by Passive Devices – a Review of Some Recent Developments. In: Gyr, A. (ed.) Structure of Turbulence and Drag Reduction. International Union of Theoretical and Applied Mechanics. Springer, Berlin (1990)

[29] Savill AM, Mumford JC (1988). Manipulation of turbulent boundary layers by outer-layer devices: Skin-friction and flow-visualization results. J. Fluid Mech..

[30] Kim JS, Hwang J, Yoon M, Ahn J, Sung HJ (2017). Influence of a large-eddy breakup device on the frictional drag in a turbulent boundary layer. Phys. Fluids.

[31] Spalart PR, Strelets M, Travin A (2006). Direct numerical simulation of large-eddy-break-up devices in a boundary layer. Int. J. Heat Fluid Flow.

[32] Anders, J.B.: Boundary layer manipulators at high Reynolds numbers. In: Gyr, A. (ed.) Structure of turbulence and drag reduction IUTAM symposium Zurich/Switzerland 1989, pp. 475–482. Springer (1990)

[33] Monkewitz PA, Chauhan KA, Nagib HM (2007). Self-consistent high-Reynolds-number asymptotics for zero-pressure-gradient turbulent boundary layers. Phys. Fluids.

[34] Monkewitz PA, Nagib HM (2015). Large-reynolds-number asymptotics of the streamwise normal stress in zero-pressure-gradient turbulent boundary layers. J. Fluid Mech..

[35] Schlatter P, Li Q, Brethouwer G, Johansson AV, Henningson DS (2010). Simulations of spatially evolving turbulent boundary layers up to re𝜃 = 4300. Int. J. Heat Fluid Flow.

[36] Bertelrud, A., Drougge, G., Landahl, M.T.: Drag reduction through boundary layer control - a report on EUROMECH 81. FFA TN 1984–60 (1984)

[37] Vernet JA, Örlü R, Alfredsson PH (2015). Separation control by means of plasma actuation on a half cylinder approached by a turbulent boundary layer. J. Wind Eng. Ind. Aerodyn..

[38] Choi KS, Hamid S (1991). Report on the 5th European drag reduction working meeting. ERCOFTAC Bulletin.

[39] Reynolds RT, Hayden P, Castro IP, Robins AG (2007). Spanwise variations in nominally two-dimensional rough-wall boundary layers. Exp. Fluids.

[40] Smith, A.E., Gordeyev, S.: Aero-optical mitigation of turbulent boundary layers using large-eddy break-up devices AIAA paper 2014–0321 (2014)

[41] Choi KS, Yang X, Clayton BR, Glover EJ, Atlar M, Semenov BN, Kulik VM (1997). Turbulent drag reduction using compliant surfaces. Proc. r. Soc. Lond. A.

[42] Bird, J., Santer, M., Morrison, J.F.: Experimental control of turbulent boundary layer with in-plane forcing. flow, Turbulence Combust (submitted) (2018) 10.1007/s10494-018-9926-2 PMC6044235 30069149

[43] Choi KS, Clayton BR (2001). The mechanism of turbulent drag reduction with wall oscillation. Int. J. Heat Fluid Flow.

[44] Sattarzadeh SS, Fransson JHM, Talamelli A, Fallenius BE (2014). Consecutive turbulence transition delay with reinforced passive control. Phys. Rev. E.

[45] Vernet, J.A., Örlü, R., Söderblom D., Elofsson, P., Alfredsson, P.H.: Plasma streamwise vortex generators for flow separation control on trucks – A proof-of-concept experiment. In Print (2018) 10.1007/s10494-018-9891-9 PMC6044254 30069150

[46] Lundell F (2007). Reactive control of free-stream turbulence induced transition: an experimental demonstration. J. Fluid Mech..

